# The Prognostic and Clinicopathological Roles of Sirtuin-3 in Various Cancers

**DOI:** 10.1371/journal.pone.0159801

**Published:** 2016-08-02

**Authors:** Fei-Yuan Yu, Qian Xu, Dan-Dan Wu, Andy T. Y. Lau, Yan-Ming Xu

**Affiliations:** 1 Laboratory of Cancer Biology and Epigenetics, Department of Cell Biology and Genetics, Shantou University Medical College, Shantou, Guangdong, 515041, P. R. China; 2 Department of Pathology, Shantou University Medical College, Shantou, Guangdong, 515041, P. R. China; China Medical University, TAIWAN

## Abstract

Sirtuin-3 (SIRT3) is a major mitochondrial NAD(+)-dependent deacetylase and plays a key role in the progression and development of human cancers. Although the prognostic and clinicopathological features of SIRT3 expression in various cancers have been investigated by different research groups, however, inconsistent and opposing results can be observed. In this study, we therefore performed a meta-analysis to evaluate the significance of SIRT3 expression in various cancers. Systematic literature searching was performed in PubMed, Embase, China National Knowledge Infrastructure, and Wanfang Data up to November 2015. Total effect analyses and subgroup analyses were performed to evaluate the relationship between SIRT3 expression and overall survival, cancer/non-cancer tissues, lymph node metastasis, pathological differentiation, tumor node metastasis (TNM) stage, tumor size, and gender, in various cancer patients. Hazard ratios (HRs) or odds ratios (ORs) with 95% confidence intervals (CIs) were calculated to clarify the risk or hazard association. A total of 14 studies comprising 2165 cancer patients were included to assess the association between SIRT3 immunohistochemical expression and overall survival or clinicopathological characteristics. SIRT3 expression was significantly associated with overall survival in gastric cancer (HR = 0.62, 95% CI = 0.43–0.89, P = 0.009) and hepatocellular carcinoma patients (HR = 0.56, 95% CI = 0.42–0.74, P<0.0001), cancer/non-cancer tissues in hepatocellular carcinoma patients (OR = 0.04, 95% CI = 0.01–0.16, P<0.0001), lymph node metastasis in breast cancer patients (OR = 2.20, 95% CI = 1.49–3.26, P<0.0001), and also pathological differentiation in hepatocellular carcinoma patients (OR = 0.69, 95% CI = 0.48–0.98, P = 0.04) and gastric cancer patients (OR = 0.33, 95% CI = 0.21–0.50, P<0.00001), by subgroup analyses. Furthermore, SIRT3 expression was significantly associated with pathological differentiation in total effect analysis (OR = 0.46, 95% CI = 0.29–0.74, P = 0.001). No detectable relation between SIRT3 expression and other clinicopathological parameters were found. This meta-analysis indicates that SIRT3 expression level is associated with prognostic and clinical features in specific cancers.

## Introduction

The sirtuin (SIRT) family consists of seven members, SIRT1-SIRT7, which are evolutionarily conserved proteins that function as NAD(+)-dependent deacetylases or ADP-ribosyltransferases in eukaryotes [1]. SIRTs, in addition to regulate multiple aspects of physiological responses including cell stress and metabolism [2], play key roles in aging [3] and cardiac disease [4].

SIRT3, a genomically-expressed, mitochondrial-localized member of SIRT family, is critical for maintaining mitochondrial integrity and function by directing multiple metabolic processes through deacetylating numerous downstream protein substrates [5]. There have been concerns in recent years due to SIRT3’s emerging roles in cancer by regulating both cell death and survival. SIRT3 was thought to participate a wide range of cancerous functions, including genomic instability and mutation, resisting cell death, sustaining proliferative signaling, deregulating cellular energetics, evading growth suppressors, as well as tumor-promoting inflammation [6, 7].

Owing to its important role in cancers, the clinical relevance of SIRT3 expression to cancer progression and prognosis was noticed by the research community. In recent years, the association between SIRT3 expression and clinicopathological parameters were evaluated in patients with breast cancer, colon cancer, esophageal cancer, gastric cancer, hepatocellular carcinoma, oral squamous cell carcinoma, prostate cancer, pancreatic cancer, thyroid carcinoma, as well as head and neck cancer. Expression of SIRT3 was reported to be significantly associated with poor prognosis in cancers such as breast cancer [8], colon cancer [9], and esophageal cancer [10]; but opposite results were reported in hepatocellular carcinoma [11, 12] and gastric cancer [13, 14]. The correlation between SIRT3 expression and other clinical outcomes such as pathological differentiation across different cancers also remains controversial. Zhang et al. [12] reported that SIRT3 was correlated with differentiation in hepatocellular carcinoma while opposite result was reported by Wang et al. [11]. Up to date, there are still no systematic reviews or meta-analyses discussing the role and clinical significance of SIRT3 in cancers. In this study, we therefore intend to pool and analyze clinical data reported from multiple research studies in order to provide a complete, exhaustive summary of current literatures relevant to the prognostic and clinicopathological roles of SIRT3 in various human cancers.

## Materials and Methods

### Literature search

Systematic literature searching was performed in PubMed, Embase, China National Knowledge Infrastructure (CNKI), and Wanfang Data. The search ceased on November 9, 2015. We used the search terms: “SIRT3” OR “SIRT 3” OR “SIRT-3” OR “sirtuin3” OR “sirtuin 3” OR “sirtuin-3” OR “SIR2L3” OR “silent mating type information regulation 2 homolog 3” AND “cancer” OR “tumor” OR “neoplasm” OR “carcinoma”.

### Inclusion and exclusion criteria

The inclusion criteria for primary studies were as follows: (1) human studies; (2) SIRT3 expression was detected by immunohistochemistry (IHC); and (3) SIRT3 expression was reported. The following exclusion criteria for published studies were used: (1) reviews, conference abstracts, and case reports; (2) cell or animal studies; (3) no description of detection methods; (4) a lack of SIRT3 expression data or the IHC results were only reported in figures; (5) the HRs and 95% CIs of survival analyses were not reported or could not be calculated or extracted from other data; and (6) all articles with the data from the same patient population, except for the most recent report.

### Data extraction

Data from appropriate studies were independently extracted by two investigators using the same criteria. The major items included the following: first author’s name, year of publication, study location, cancer type, total cases, SIRT3 assessment methods, cut-off definition, data of SIRT3 overexpression for overall survival, and clinicopathological parameters (cancer/non-cancer, lymph node metastasis, pathological differentiation, and tumor stage, etc.).

### Statistical methods

Statistical analysis was carried out using the Cochrane Collaboration Review Manager 5.3 software (Cochrane Collaboration, Copenhagen, Denmark) in this meta-analysis. For time-to-event analyses, the association between SIRT3 and overall survival (OS), HR and 95% CI were combined to estimate the effect. Survival data was directly extracted or indirectly extracted as described by Tierney et al. [15]. For dichotomous data, such as expression of SIRT3 and clinicopathological features including lymph node metastasis, gender, tumor size, tumor stage and pathological differentiation, ORs and 95% CIs were combined to estimate the effect. I^2^ statistics and χ^2^ test were used to evaluate the heterogeneity among analyses in this meta-analysis. Random effects model was used when the heterogeneity was significant between studies (I^2^>50% or P<0.10); otherwise, the fixed effects model was used. The significance of the pooled HR or OR was determined by Z test, and P<0.05 was considered statistically significant. The potential publication bias was evaluated by executing funnel plot. An asymmetric plot indicates there is potential publication bias; otherwise, the plot should shape like a funnel.

## Results

### Eligible studies and characteristics

**Fig 1:**
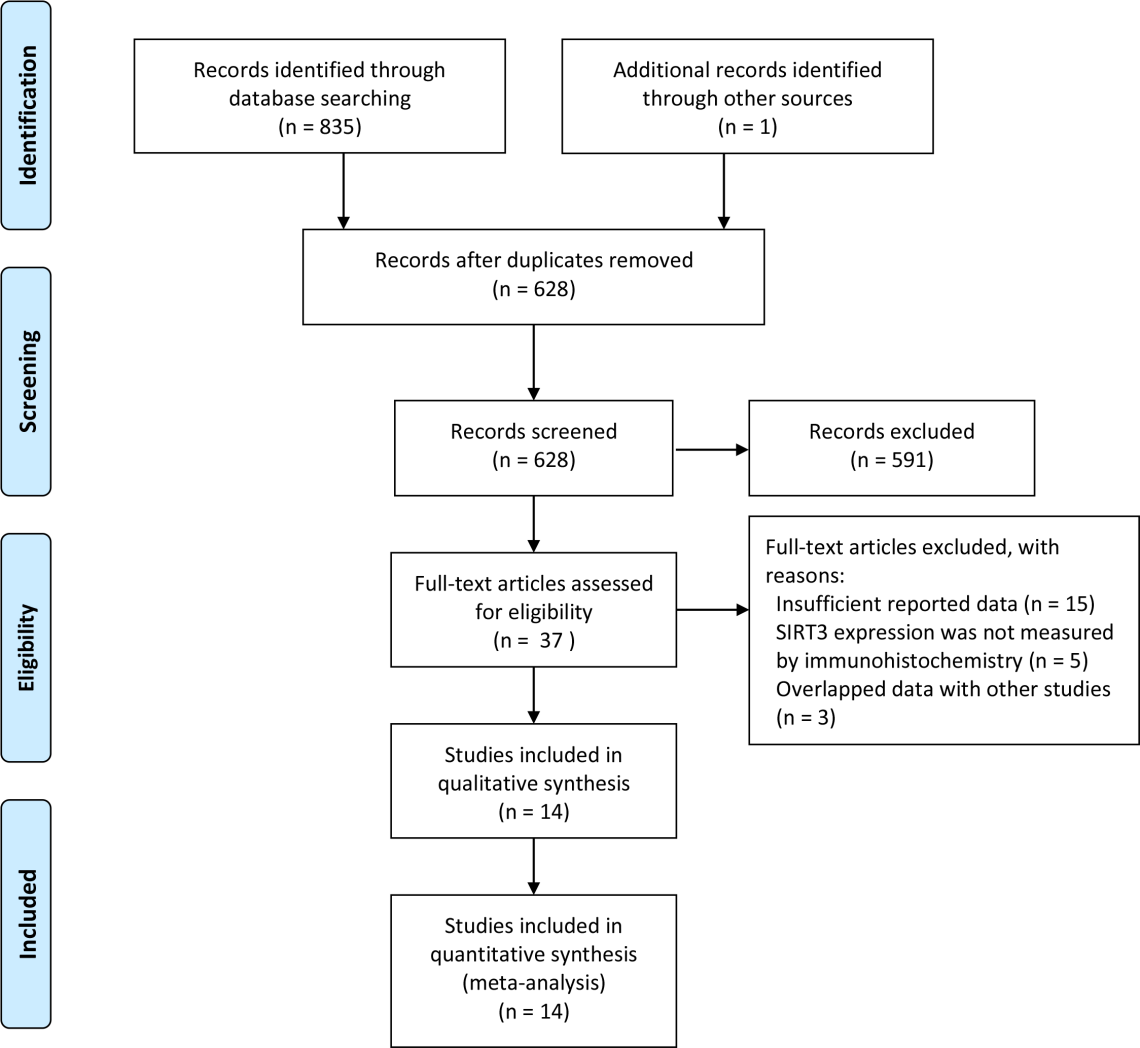
Flow diagram of identified eligible studies.

Search strategy for eligible studies was described in Fig 1. According to our retrieval strategy, a total of 836 articles were identified. After redundant articles were removed, 591 studies were excluded by title and abstract review which did not meet the selection criteria. Upon further reviewing, 23 studies were excluded for the following reasons: (1) insufficient reported data (n = 15); (2) SIRT3 expression was not measured by immunohistochemistry (n = 5); and (3) Data overlapped with other studies (n = 3). Finally, 14 studies with pathological results and clinical data were included.

As summarized in Table 1 and S1 Table, the 14 eligible studies include 12 English articles and 2 Chinese articles (12 studies evaluated Asians and 2 evaluated Caucasian). These studies include 2165 patients, 7 types of cancer: breast cancer, esophageal squamous cell carcinoma, colon cancer, gastric cancer, hepatocellular carcinoma, oral squamous cell carcinoma, and prostate cancer. Immunohistochemistry was used to detect the expression level of SIRT3 in all these studies.

**Table 1:** Main characteristics of studies included in this meta-analysis.

Study [ref. no.]	Year	Country/region	Race	Cancer type	No. of cases	Detection method	Cut off	Outcome
Desouki et al. [5]	2014	USA	Caucasian	Breast cancer	186	IHC	≥1%	NA
He et al. [8]	2014	China	Asian	Breast cancer	308	IHC	IRS>6	OS
Liu et al. [9]	2014	China	Asian	Colon cancer	127	IHC	IRS≥6	OS
Yan et al. [10]	2014	China	Asian	Esophageal squamous cell carcinoma	252	IHC	IRS>6	OS
Yang et al. [13]	2014	China	Asian	Gastric cancer	65	IHC	HS≥10%	OS
Huang et al. [14]	2014	Taiwan	Asian	Gastric cancer	221	IHC	1+	OS
Kang [29]	2014	China	Asian	Gastric cancer	130	IHC	1+	NA
Cui et al. [30]	2015	China	Asian	Gastric cancer	43	IHC	NA	NA
Wang et al. [11]	2014	China	Asian	Hepatocellular carcinoma	342	IHC	≥5%	OS
Zhang et al. [12]	2012	China	Asian	Hepatocellular carcinoma	248	IHC	ROC≥2.5	OS
Zhang et al. [31]	2013	China	Asian	Hepatocellular carcinoma	40	IHC	1+	NA
Liu [32]	2012	China	Asian	Hepatocellular carcinoma	42	IHC	1+	NA
Alhazzazi [33]	2012	USA	Caucasian	Oral squamous cell carcinoma	52	IHC	NA	NA
Quan et al. [34]	2015	China	Asian	Prostate cancer	109	IHC	NA	NA

IHC, immunohistochemistry; IRS, immunoreactivity scores; HS, histoscore method; ROC, receiver-operator characteristic; NA, not available; OS, overall survival.

### SIRT3 expression and OS in patients with cancers

**Fig 2:**
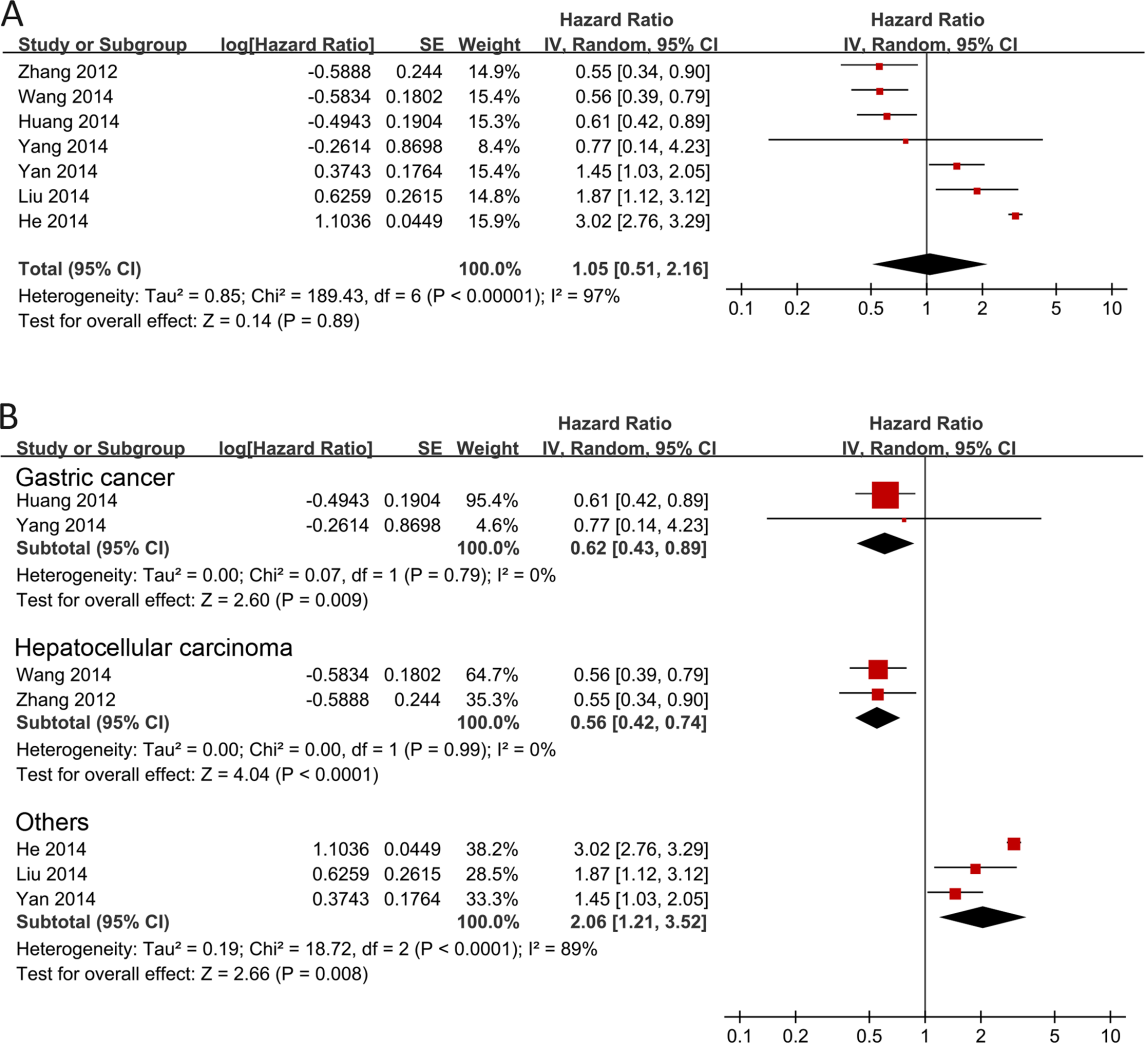
Forest plot for the association between SIRT3 expression and overall survival of patients with cancer.

In the 7 studies with survival data, there was obvious heterogeneity (I^2^ = 97.0%). Thus, a random effects model was used to calculate the pooled HR with corresponding 95% CI. The meta-analysis found that there was no detectable relation between SIRT3 expression and prognosis in various cancer patients with the pooled HR of 1.05 (95% CI = 0.51–2.16, P = 0.89) (Fig 2A). Due to the high heterogeneity (I^2^ = 97.0%) in the meta-analysis, it is necessary to perform subgroup analyses by cancer types to explore the between-study heterogeneity. Subgroup analyses by cancer types suggested that there was obviously significant association between SIRT3 expression and OS in gastric cancer (HR = 0.62, 95% CI = 0.43–0.89, P = 0.009) and hepatocellular carcinoma (HR = 0.56, 95% CI = 0.42–0.74, P<0.0001) patients, which indicated patients with higher SIRT3 expression have longer overall survival (Fig 2B). On the other hand, opposite results (HR = 2.06, 95% CI = 1.21–3.52, P = 0.008) were observed in the pooled group of studies of Asian patients with breast cancer, colon cancer, and esophageal squamous cell carcinoma (Fig 2B, Others).

### Correlation of SIRT3 expression with clinicopathological parameters

Total effect analyses and subgroup analyses (by cancer type) were used to assess the association between SIRT3 expression and clinicopathological parameters in various cancer patients (Table 2). A meta-analysis including eight studies with SIRT3 expression data in cancer/non-cancer tissues demonstrated that there was no correlation between SIRT3 expression in cancer and non-cancer tissues (OR = 0.34, 95% CI = 0.12–0.99, P = 0.05). Significant differences between SIRT3 positivity/high expression and cancer/non-cancer tissues were found in hepatocellular carcinoma (OR = 0.04, 95% CI = 0.01–0.16, P<0.0001) through subgroup analyses, suggested that expression of SIRT3 was downregulated in hepatocellular carcinoma tissues as compared with noncancerous tissues. Besides the above parameters mentioned, we also performed meta-analyses to evaluate the correlation between SIRT3 expression and controversial parameters such as lymph node metastasis and pathological differentiation. The meta-analysis suggested that SIRT3 overexpression was also not correlated with lymph node metastasis in total effect analyses (OR = 1.64, 95% CI = 0.80–3.39, P = 0.18). However, in subgroup analyses, for lymph node metastasis of breast cancer in the SIRT3 positivity/high group, as compared with the SIRT3 negative/low group, was statistically significant (OR = 2.20, 95% CI = 1.49–3.26, P<0.0001). Results obtained from seven studies which evaluated the relationship between SIRT3 expression and pathological differentiation in cancer patients, yielding a pooled OR of 0.46 (95% CI = 0.29–0.74, P = 0.001), indicating there is significant correlation between SIRT3 expression and pathological differentiation. In subgroup analyses, patients with lower SIRT3 expression correlate with poor differentiation as compared with those with higher SIRT3 expression in hepatocellular carcinoma (OR = 0.69, 95% CI = 0.48–0.98, P = 0.04) and gastric cancer (OR = 0.33, 95% CI = 0.21–0.50, P<0.00001). In addition, no significant correlation was found between the expression of SIRT3 and tumor stage (OR = 1.68, 95% CI = 0.85–3.25, P = 0.12), tumor size (OR = 0.85, 95% CI = 0.60–1.22, P = 0.38), gender (OR = 1.06, 95% CI = 0.79–1.41, P = 0.71), estrogen receptor (ER) (OR = 1.92, 95% CI = 0.95–3.90, P = 0.07) and progesterone receptor (PR) (OR = 0.95, 95% CI = 0.22–4.10, P = 0.95) in total or subgroup analyses (Table 2).

**Table 2:** Correlations of clinicopathological parameters with SIRT3 expression.

Parameters	Total effect or subgroup analyses	Studies [ref. no.]	Heterogeneity		Model	Outcomes	
			I^2^%	P value		OR (95% CI)	P value
Cancer/non-cancer tissues	Total	[5, 13, 29–34]	83	<0.00001	Random	0.34 (0.12, 0.99)	0.05
	Gastric cancer	[13, 29, 30]	91	<0.0001	Random	0.97 (0.12, 7.89)	0.98
	Hepatocellular carcinoma	[31, 32]	0	0.96	Fixed	0.04 (0.01, 0.16)	<0.0001
	Others	[5, 33, 34]	85	0.001	Random	0.43 (0.08, 2.44)	0.34
Lymph node metastasis	Total	[5, 8, 9, 13, 29]	78	0.001	Random	1.64 (0.80, 3.39)	0.18
(metastasis+ vs. metastasis-)	Breast cancer	[5, 8]	34	0.22	Fixed	2.20 (1.49, 3.26)	<0.0001
	Gastric cancer	[13, 29]	79	0.03	Random	0.69 (0.13, 3.62)	0.66
	Other	[9]	-	-	-	4.22 (2.00, 8.93)	0.0002
Differentiation (poorly vs. moderate/well differentiated)	Total	[11–14, 29, 31, 32]	57	0.03	Random	0.46 (0.29, 0.74)	0.001
	Gastric cancer	[13, 14, 29]	50	0.13	Fixed	0.33 (0.21, 0.50)	<0.00001
	Hepatocellular carcinoma	[11, 12, 31, 32]	4	0.37	Fixed	0.69 (0.48, 0.98)	0.04
Tumor stage (I/II vs. III/IV)	Total	[9, 10, 12–14, 29]	82	<0.0001	Random	1.68 (0.87, 3.25)	0.12
	Gastric cancer	[13, 14, 29]	86	0.0007	Random	3.09 (0.76, 12.54)	0.11
	Others	[9, 10, 12]	82	0.004	Random	1.09 (0.49, 2.42)	0.84
Tumor size (≥5cm vs. <5cm)	Total	[9, 12, 29, 31, 32]	0	0.93	Fixed	0.85 (0.60, 1.22)	0.38
	Hepatocellular carcinoma	[12, 31, 32]	0	0.89	Fixed	0.81 (0.51, 1.29)	0.38
	Others	[9, 29]	0	0.48	Fixed	0.92 (0.53, 1.60)	0.76
Gender (male vs. female)	Total	[9–13, 29, 31, 32]	12	0.33	Fixed	1.06 (0.79, 1.41)	0.71
	Gastric cancer	[13, 29]	0	0.34	Fixed	1.66 (0.78, 3.55)	0.19
	Hepatocellular carcinoma	[11, 12, 31, 32]	0	0.82	Fixed	1.32 (0.84, 2.08)	0.22
	Others	[9, 10]	31	0.23	Fixed	0.73 (0.47, 1.13)	0.16
ER (positive vs. negative)	Breast cancer	[5, 8]	66	0.09	Random	1.92 (0.95, 3.90)	0.07
PR (positive vs. negative)	Breast cancer	[5, 8]	92	0.0004	Random	0.95 (0.22, 4.10)	0.95

OR, odds ratio; CI, confidence interval; ER, estrogen receptor; PR, progesterone receptor.

### Publication bias

**Fig 3:**
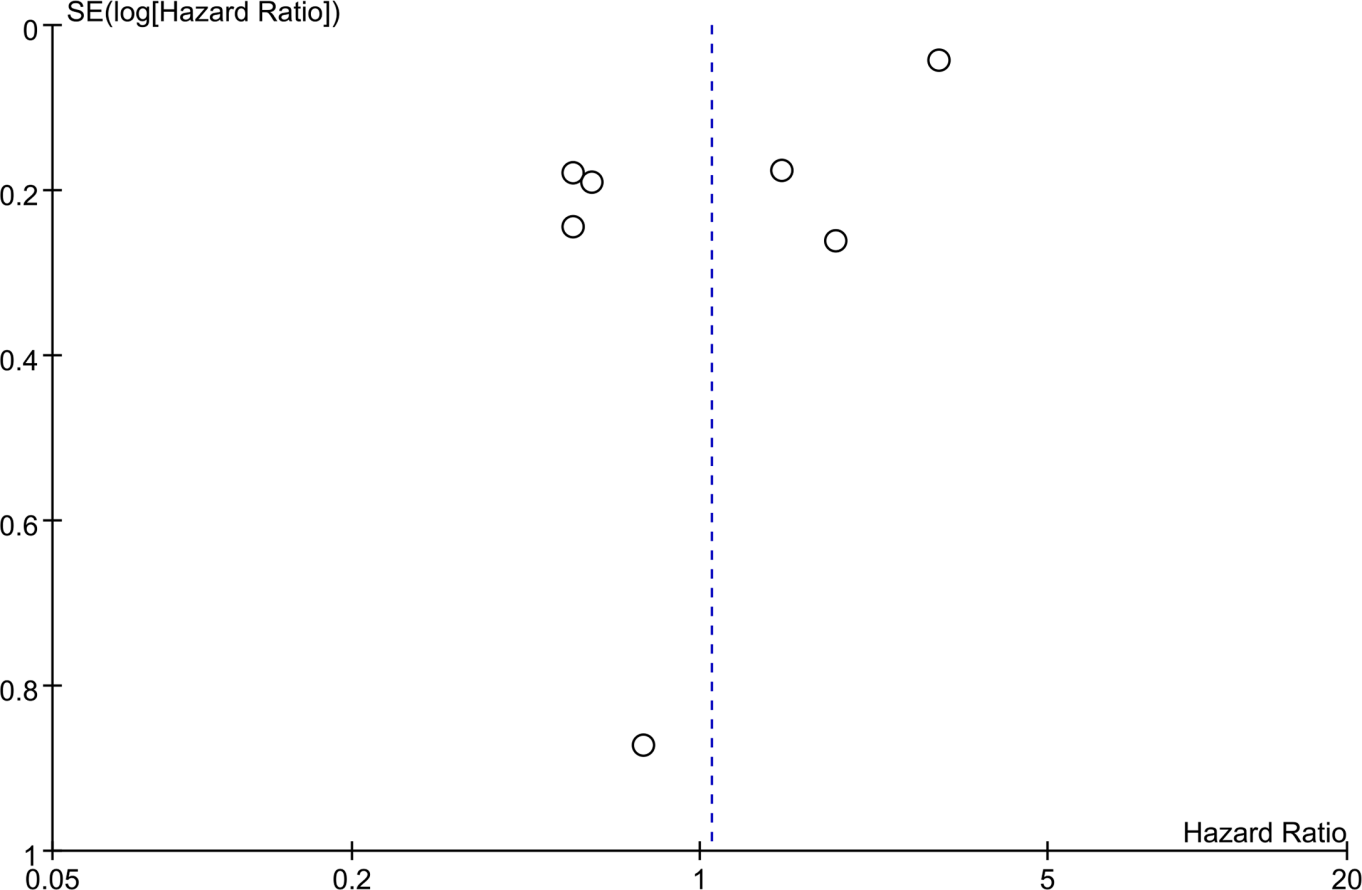
Funnel plot for the seven studies included in this meta-analysis regarding SIRT3 expression and overall survival.

Publication bias of OS in this meta-analysis was evaluated by funnel plot analysis. No evidence of obvious asymmetry was observed in the funnel plot (Fig 3).

## Discussion

SIRT3, a major mitochondrial NAD(+)-dependent deacetylase (expressed extensively in human, mouse, zebrafish, and yeast), plays an important role in targeting mitochondrial proteins for lysine deacetylation and also regulating diverse cellular functions [7]. Up to date, oncogenic and tumor-suppressive roles of SIRT3 were observed in cancers [7].

The association between SIRT3 expression and clinical outcomes has been investigated in recent years, but no compatible results have been achieved. This is the first meta-analysis focused on the association between SIRT3 expression and patient clinicopathological outcomes including survival, cancer/non-cancer tissues, lymph node metastasis, pathological differentiation, tumor stage, tumor size, and gender. The present study pooled the survival data of 1563 cancer patients from 7 studies and found that SIRT3 overexpression was not associated with OS in various cancer patients (HR = 1.05, 95% CI = 0.51–2.16, P = 0.89), but the subgroup analyses grouped by cancer types revealed that the overexpression of SIRT3 was associated with better overall survival in hepatocellular carcinoma (HR = 0.56, 95% CI = 0.42–0.74, P<0.0001) and gastric cancer (HR = 0.62, 95% CI = 0.43–0.89, P<0.0001) patients. This might indicate the association of SIRT3 expression and overall survival in cancer patients was depended on the cancer type. To address the possibility of whether the association of SIRT3 expression and clinicopathological outcomes were cancer type specific, we further investigated the association of SIRT3 and other clinical parameters using total effect analyses and subgroup analyses grouped by cancer types. In total effect analyses, data from 8 studies showed that there was no correlation between SIRT3 expression and cancer tissues, compared with non-cancer (adjacent and/or benign) tissues (OR = 0.34, 95% CI = 0.12–0.99, P = 0.05), and no significant correlation was found between SIRT3 expression and lymph node metastasis in total analyses. On the contrary, subgroup analyses revealed that SIRT3 expression was significantly decreased in hepatocellular carcinoma tissues (OR = 0.04, 95% CI = 0.01–0.16, P<0.0001) as compared with noncancerous tissues, and upregulation of SIRT3 is a risk factor for lymph node metastasis in breast cancer patients (OR = 2.20, 95% CI = 1.49–3.26, P<0.0001). Furthermore, in the 4 studies with pathological differentiation data in hepatocellular carcinoma patients, a significant association was found between SIRT3 expression and pathological differentiation (OR = 0.69, 95% CI = 0.48–0.98, P = 0.04). These findings strengthen the notion that the function of SIRT3 may be diverse depending on the cell-type or tumor-type, which was in agreement with the viewpoint of Chen et al. [7]. These results also indicate that SIRT3 expression may be associated with the observed heterogeneity of various cancers in this meta-analysis [7].

Information on tumor stage, tumor size, gender, and positive/negative of ER or PR expression (as in breast cancer), all of which are important clinical characteristics correlated with tumor progression, were extracted and analyzed in this meta-analysis. Yet, no correlation was observed between SIRT3 expression and these clinicopathological features.

The mechanism for the apparent relationship in prognostic and clinical features associated with SIRT3 expression remains uncertain. In certain type of cancers, the participation of SIRT3 protein in hormonal regulation may be a potential contributing factor. In 2010, Kim et al. reported that SIRT3 knockout mice developed ER-positive mammary tumors [16]. In addition, reduced expression of SIRT3 was observed in human breast cancer [17]. In this meta-analysis, we found that SIRT3 was downregulated in hepatocellular carcinoma tissues as compared with noncancerous tissues, which is in accord with the report of Finley et al [17]. Several properties of SIRT3 may contribute to the outcome association in patients with cancer. The ability of SIRT3 to suppress tumor growth via inhibiting reactive oxygen species(ROS) production and regulating HIF-1α stabilization of host cells has attracted considerable attention in recent years [18–20]. In the aspect of liver cancer, to the best of our knowledge, viral hepatitis (hepatitis B virus and hepatitis C virus in particular) is an important reason of the cause of liver cancer [21, 22]. After viral infection, a proportion of patients will progress to the development of chronic hepatitis and result in liver cancer [23], as well as fulminant hepatitis. Viral hepatitis, as a possible initiating event of liver cancer, will significantly induce oxidative stress [24]. Oxidative stress contributes to genotoxicity through the effect of ROS on diverse cellular processes, which will promote DNA damage and genetic alterations that are strongly implicated in the initiation and/or early events that lead to carcinogenesis [25]. SIRT3 can regulate the enzymatic activity of MnSOD to maintain the balance of ROS, attributing its tumor-suppression effect [20]. Another mechanism for the tumor-suppression effect of SIRT3 in liver cancer is that SIRT3 could decrease ROS induced by hepatitis B virus x protein (HBx) after viral infection [26]. The feature of SIRT3 in oxidative stress regulation may also strongly benefit in the management of fulminant hepatitis caused by viruses, drugs, or toxic agents [27]. Therefore, SIRT3 contributes in maintaining the balance of ROS in host cells and attenuating organ injury. These results indicate that SIRT3 function as a tumor suppressor in the initiation and/or early events of carcinogenesis. In addition, SIRT3 exerts its tumor suppressive function by repressing ERK1/2, increasing Bax and/or activating caspase-3 [7]. The tumor suppressor role of SIRT3 suggests that SIRT3 might correlate with the development of the pathological feature in cancers, which was observed in our result that patients with higher SIRT3 expression correlate with well/moderate differentiation as compared with those with lower expression in hepatocellular carcinoma and gastric cancer. On the contrary, our results demonstrate that upregulation of SIRT3 was a risk factor for lymph node metastasis in breast cancer patients, which implicate the tumor promoter role of SIRT3 in advanced stages of breast cancer. Furthermore, the prognostic finding that patients with higher expression of SIRT3 had shorter overall survival in our meta-analysis also reveal that SIRT3 function as tumor promoter after cells have been completely transformed. The mechanism for SIRT3 as a tumor promoter can be explained by the fact that SIRT3 might engage in cross-talk with Fas/receptor-interacting protein (RIP)/focal adhesion kinase (FAK) death–survival pathways in cancer cells [7]. In addition, acetylation of nicotinamide mononucleotide adenylyltransferase 2 (NMNAT2), decreased Tam-induced apoptosis and/or increased Ku70-Bax interaction may also contribute to the tumor promoter role of SIRT3 [7]. Up to date, most studies have shown that SIRT3 is localized in the mitochondria, but additional studies reveal that SIRT3 may also be localized in the nucleus [7]. Therefore, in specific cancers or cellular events, the shuttling and interaction of SIRT3 with various substrates in different subcellular localization might be responsible for the dual role of SIRT3. The regulation of SIRT3-related signaling in cancer processes implicate that SIRT3 might be a potential therapeutic target in cancer management [7, 28], but the obvious Janus-faced role of SIRT3 suggests that different treatment strategies should be employed in various cancer developmental stages in order to achieve better therapeutic efficacy.

There are several limitations in this study, because of our stringent criteria in the selection of eligible studies to be analyzed, which should be acknowledged. Firstly, except Asian patients, only two studies focused on Caucasian patients. It inevitably made it difficult to draw a firm conclusion on the prognostic value of SIRT3 for Caucasian and cancer patients of other races. Secondly, selection methods of cancer and non-cancer tissue samples were varied among different studies. Some were collected from different areas of the same patient and some were collected from individual patients. Thirdly, the amount of studies in some types of cancer were low, the associated biases might be remarkable due to insufficient sample size. Finally, due to lack of available data, the association between SIRT3 and other important clinical parameters such as tumor infiltration and recurrence were not able to be explored.

In conclusion, the present meta-analysis indicates that overexpression of SIRT3 is not related to overall survival in various cancer patients and there exists no correlation between SIRT3 overexpression and other clinicopathological parameters such as lymph node metastasis in total effect analyses. However, correlation is found by subgroup analyses in a certain type of cancers, which indicates that the expression and function of SIRT3 are cancer type specific. It is anticipated that well-designed clinical studies with larger number of cancer cases (with more comprehensive human races covered also) would be performed to update our viewpoints in the future.

## Supporting Information

S1 File PRISMA Checklist. (DOC)

S1 Table Data extraction table. (XLSX)
